# A simple algorithm for the treatment of traumatic cardiac arrest

**DOI:** 10.1186/1757-7241-21-S1-S10

**Published:** 2013-05-28

**Authors:** DJ Lockey, RM Lyon, GE Davies

**Affiliations:** 1 London’s Air Ambulance, London, UK

## Background

Major trauma is the leading causing of death in young adults across the globe. The mortality from traumatic cardiac arrest remains high but survival with good neurological outcome from cardiopulmonary arrest following major trauma has now been reported. Rapid, effective intervention is required to address potential reversible causes of traumatic cardiac arrest if the victim is to survive. There is no standard treatment algorithm for traumatic cardiac arrest. We present a simple algorithm to manage the major trauma patient in actual or near cardiac arrest.

## Methods

We reviewed current pre-hospital clinical practice and the published literature on major trauma management. An algorithm was developed and used regularly by London’s Air Ambulance pre-hospital physician/paramedic trauma team.

## Results

The algorithm addresses the need treat potential reversible causes of traumatic cardiac arrest. This includes immediate resuscitative thoracotomy in cases of penetrating chest or abdominal trauma resulting in cardiac arrest, airway management, optimising oxygenation, reversal of hypovolaemia using intravenous/intraosseous fluid replacement and chest decompression to exclude tension pneumothorax.

## Conclusion

A standard approach to traumatic cardiac arrest is feasible. Use of a treatment algorithm can rapidly, simultaneously address reversible causes of traumatic cardiac arrest and has the potential to save lives.

